# The prediction of postoperative delirium with the preoperative bispectral index in older aged patients: a cohort study

**DOI:** 10.1007/s40520-023-02408-9

**Published:** 2023-05-19

**Authors:** Lin Bao, Taotao Liu, Zhenzhen Zhang, Qian Pan, Lifang Wang, Guohui Fan, Zhengqian Li, Yiqing Yin

**Affiliations:** 1 Department of Anesthesiology, Peking University Third Hospital, Beijing, China; 2 Beijing Center of Quality Control and Improvement on Clinical Anesthesia, Beijing, China; 3 Department of Anesthesiology, Capital Medical University Affiliated Beijing Shijitan Hospital, Beijing, China; 4 Department of Anesthesiology, Tianjin Medical University Cancer Institute and Hospital, National Clinical Research Center for Cancer, Tianjin, China; 5 Key Laboratory of Cancer Prevention and Therapy, Tianjin, China; 6 Tianjin’s Clinical Research Center for Cancer, Tianjin, China; 7 Department of Anesthesiology, China–Japan Friendship Hospital, Beijing, China; 8 Institute of Clinical Medical Sciences, China–Japan Friendship Hospital, Beijing, China

**Keywords:** BIS, Preoperative predictive score, Postoperative delirium, Monitoring, Elderly patients, Prediction

## Abstract

**Background:**

Postoperative delirium (POD) is the most common postoperative complication in elderly patients, especially in older aged patients (aged 75 years or over). The development of electroencephalography analysis could provide indicators for early detection, intervention, and evaluation. If there are pathophysiological changes in the brain, the BIS value will also change accordingly. In this study, we investigated the predictive value of the preoperative bispectral (BIS) index in POD for patients aged over 75 years.

**Methods:**

In this prospective study, patients (≥ 75 years) undergoing elective non-neurosurgery and non-cardiac surgery under general anesthesia were included (*n* = 308). Informed consent was obtained from all involved patients. Before the operation and during the first 5 postoperative days, delirium was assessed with the confusion assessment method by trained researchers twice every day. Thereafter, the preoperative bedside BIS of each patient was dynamically acquired by the BIS VISTA monitoring system and the BIS monitoring of electrodes. A series of evaluation scales were assessed before and after surgery. A preoperative predictive score was generated according to the results of multivariable logistic regression. The receiver operating characteristic curves were drawn and the area under the curves was estimated to evaluate the perioperative diagnostic values of BIS and preoperative predictive score for POD. The specificity, sensitivity, positive predictive value (PPV), and negative predictive (NPV) value were calculated.

**Results:**

Delirium occurred in 50 of 308 (16.2%) patients. The median BIS of delirious patients was 86.7 (interquartile range [IQR] 80.0–94.0), lower than that of the non-delirious 91.9 (IQR 89.7–95.4, *P* < 0.001). According to the ROC curve of the BIS index, the optimal cut-off value was 84, with a sensitivity of 48%, specificity of 87%, PPV 43%, NPV 89% for forecasting POD and the area under curves was 0.67. While integrating BIS, mini-mental state examination, anemia, activities of daily living, and blood urea nitrogen, the model had a sensitivity of 78%, specificity of 74%, PPV of 0.37%, and NPV of 95% for forecasting POD, and the area under curves was 0.83.

**Conclusions:**

Preoperative bedside BIS in delirium patients was lower than that in non-delirium patients when undergoing non-neurosurgery and non-cardiac surgery in patients aged over 75. The model of integrating BIS, mini-mental state examination, anemia, activities of daily living, and blood urea nitrogen is a promising tool for predicting postoperative delirium in patients aged over 75.

**Supplementary Information:**

The online version contains supplementary material available at 10.1007/s40520-023-02408-9.

## Introduction

Postoperative delirium (POD) is the most common complication after surgery in the elderly, with an incidence of 11–46% in cardiac surgery and 13–50% overall in non-cardiac surgery [1]. POD is a reversible state of acute mental disorder, which will increase the postoperative mechanical ventilation time of patients and the incidence of nosocomial infection [2], prolong the hospitalization time of patients, lead to postoperative cognitive decline and increase the mortality [1, 3]. Moreover, delirium patients’ daily survival costs are twice as high as those without delirium, with delirium costing anywhere from $38 billion to $152 billion annually in America [4].

So far, there were no effective treatments for POD. Up to 40% of POD is preventable in clinical practice [5], which means early diagnosis of POD is extremely important. The Confusion Assessment Method (CAM or CAM-ICU) is used as the gold standard for delirium assessment according to the European society of anesthesiology guidelines of 2017. Some training is required to diagnose delirium with CAM or CAM-ICU. Even though CAM or CAM-ICU is used, missed diagnosis or misdiagnosis can be caused by differences in the expertise of evaluators. In a non-ICU study, 167 nurses used CAM to evaluate the delirium of 170 postoperative patients. Compared with expert evaluation, 75% of delirium patients were not evaluated correctly and the consistency of evaluation was poor (*κ* = 0.34) [6]. In a multicentric study, nurses in the ICU bedside used CAM-ICU to assess delirium in 181 patients diagnosed by a joint team of psychiatrists, geriatricians, or neurologists. The sensitivity and specificity of nurses in assessing delirium were 47% and 98% [7]. Therefore, it is essential to find an objective assessment tool to filter high-risk groups for early prevention and diagnosis.

Instead of whole-brain electroencephalography (EEG), the EEG of the frontal lobe is more convenient to assess delirium [8]. The bispectral (BIS) index is not only used for monitoring of consciousness disorders [5], but also for the diagnosis of delirium. The bilateral BIS index was lower in delirious patients compared to non-delirious and showed high specificity and low sensitivity as a predictor for early postoperative delirium after cardiac surgery in ICU [9]. In the current study, we aimed to find whether preoperative bedside BIS monitoring is a predictive value for postoperative delirium in older aged patients when undergoing non-neurosurgery and non-cardiac surgery.

## Materials and methods

### Patients

Ethical approval was obtained from the committee of the China–Japan Friendship Hospital (no. 2018-32-k23). This study was registered at chictr.org.cn (ChiCTR1800015161) and was carried out in China–Japan Friendship Hospital. All the patients received anesthesia visits one day before the surgery to fully inform the patients of the research content and answer the relevant questions. The informed consent was obtained by the patients themselves or their authorized relatives. Patients with preoperative delirium were excluded due to inability to complete basic information collection. Preoperative delirium was evaluated in the same way as POD assessment mentioned in Section “Delirium assessments”. Eligible patients were aged 75 years (or over) and undergoing non-neurosurgery and non-cardiac surgery with general anesthesia. Patients were excluded if they refused (or delirium state) or were undergone body surface surgery.

### Data collection

For patient evaluation, a preoperative questionnaire was finished, including the age, gender, body mass index (BMI), Charlson Comorbidity Index (CCI), the activity of daily living (ADL), Richards–Campbell Sleep Questionnaire (RCSQ) [10], Mini-Mental State Examination (MMSE) [11] and Geriatric Depression Scale (GDS-15) [12]. Furthermore, BIS, CAM or CAM-ICU, and the Richmond Agitation-Sedation Scale (RASS) score were noted. Additionally, the duration of surgery and intraoperative medication were recorded. Perioperative blood tests included biochemical tests, blood routine examination, etc.

### Delirium assessments

Delirium was diagnosed by two independent researchers well trained before the assessment. Delirium or non-delirium was recorded only when the assessments of two independent investigators were consistent. When researchers were uncertain regarding the evaluation of delirium, the delirium assessment was referred to a neurologist for adjudication. Patients were assessed daily at 7–8 AM and 7–8 PM on postoperative days 1 through 5 unless patients were discharged or sedated (RASS < − 3). CAM [13] scale was used for patients in general wards and the CAM-ICU [14] scale for patients with intubation in ICU.

### Bilateral bispectral index

One day before the surgery, BIS monitoring was performed using the BIS VISTA (program version 3.22) monitoring system and BIS monitoring electrodes (ASPECT Medical Systems, Norwood, MA, USA). Keep the environment quiet, and clean the forehead and bilateral temporal skin with alcohol and water. The electrodes were placed in accordance with the instructions. The researchers record the BIS index for about 5 min continuously with a signal quality index ≥ 65 and electromyography < 50. The patient lay down and closed eyes when BIS was recording. The average value of BIS data was used for statistics. BIS values were measured for 5 consecutive days after the operation and the mean values were calculated.

### Definitions

Anemia is defined as an adult male hemoglobin level less than 120 g/L or an adult female hemoglobin level of less than 110 g/L. Visual disturbance includes previous cataracts (nonsurgically treated) and visual impairment affecting daily life. The preoperative depression score was evaluated by the GDS-15 scale. The presence of depressive symptoms was defined as a GDS-15 score ≥ 8. RCSQ was used to assess sleep from five dimensions: whether it is difficult to fall asleep, the number of awakenings during the night, whether it is difficult to fall asleep again after awakening, sleep depth, and comprehensive sleep quality. Pain scores were assessed using the numeric rating scale (NRS).

### Sample size

According to a previous review, the incidence of postoperative delirium in non-cardiac surgery was about 13–50% [1], we assumed a delirium incidence of 15% in this study. Furthermore, we assumed a 10% dropout. The confidence level was 0.95 and power of 0.80. With these assumptions, 292 patients were needed based on our pilot study.

### Methods of anesthesia

General anesthesia was induced with fentanyl (0.003 mg/kg), propofol (1-2 mg/kg), and etomidate (0.1–0.2 mg/kg). Muscle relaxation was achieved using cisatracurium (0.2 mg/kg). Anesthesia was maintained with a remifentanil infusion and a propofol infusion and/or the volatile anesthetic sevoflurane. BIS was maintained between 40 and 60 during the operation. Medications used during the procedure were recorded, including anticholinergic drugs, sedatives, or dexmedetomidine. Routine management for intraoperative hypotension included reducing anesthetic depth, fluid infusion, and administration of vasopressors such as ephedrine, and/or norepinephrine. Patients were returned to ward or ICU according to their condition after operation.

### Statistical analysis

Continuous variables and categorical variables were expressed as median (interquartile range, IQR) and number (proportion), respectively. Two-group comparisons were performed by the Mann–Whitney *U* test or *χ*^2^ test, where appropriate. A preoperative predictive score was generated according to the results of multivariable logistic regression. The receiver operating characteristic (ROC) curves were drawn and the area under curves and 95% confidence intervals (CIs) were estimated to evaluate the predictive values of preoperative and diagnosis values of postoperative BIS, and preoperative predictive score for POD. The specificity, sensitivity, positive prediction value (PPV), and negative prediction value (NPV) were also calculated.

A two-sided α less than 0.05 was considered statistically significant for all statistical tests. Statistical analyses were performed by the SAS software, version 9.4 (SAS Institute Inc.) unless otherwise indicated.

## Results

**Fig. 1:**
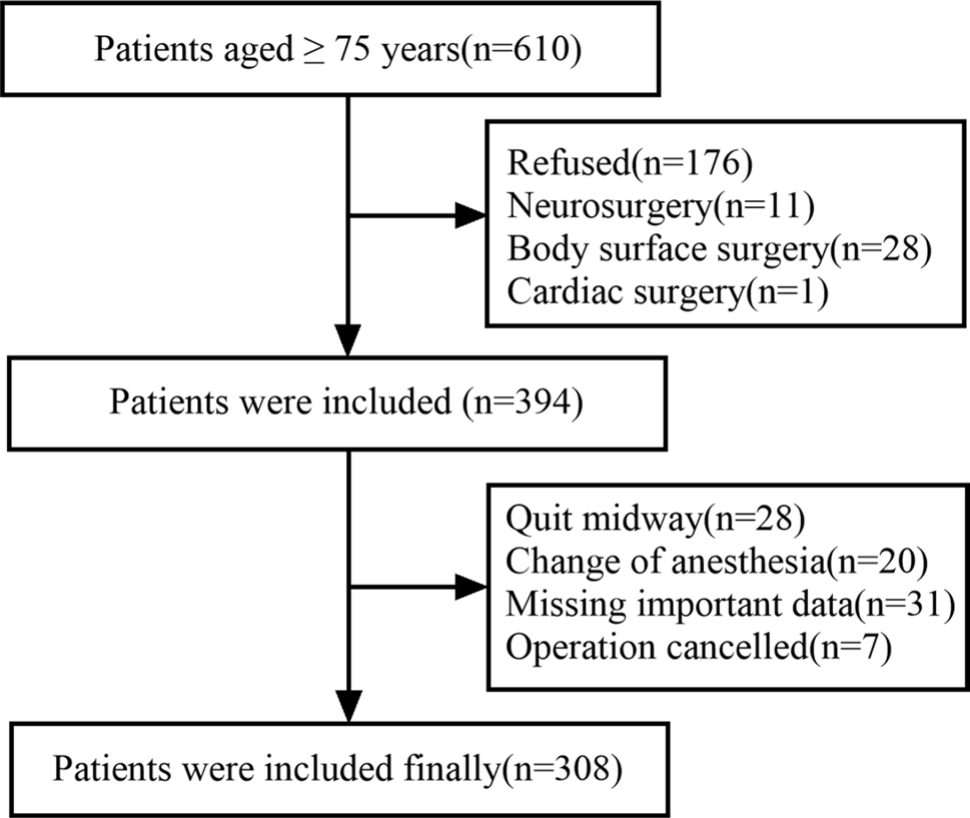
Flow chart of included patients

From April 1, 2018, to July 31, 2019, 610 patients admitted to the surgery department of China–Japan Friendship Hospital met the inclusion criteria. The operations performed include orthopedic surgery, general surgery, urological surgery, otolaryngological surgery, thoracic surgery, and gynecological surgery. At last, 394 patients were included in this study, and 308 of them were subjected to data analysis (Fig. 1).

### Patient characteristics

As shown in Table 1, there were several preoperative characteristics significantly different from the two groups (delirium *versus* non-delirium), including the age (older in the delirium group), activities of daily living score (lower in the delirium group), preoperative MMSE score (lower in the delirium group), anemia rate (higher in the delirium group), white blood cell (higher in the delirium group), blood urea nitrogen (higher in the delirium group), and albumin (lower in the delirium group). In addition, the different characteristics were as follows, discharge activity score (lower in the delirium group), transfusion rate (higher in the delirium group), the duration of anesthesia (longer in the delirium group), the postoperative hemoglobin (lower in the delirium group) and anemia rate (higher in the delirium group). There was no significant difference between groups in the types of surgery and anesthesia.

**Table 1:** Patient characteristics

Characteristics	Non-delirium (*n* = 258)	Delirium (*n* = 50)	Total (*n* = 308)	Difference* 95% CI	*P*
Gender, male	115 (44.6)	21 (42.0)	136 (44.2)	– 2.6 (– 17.5, 12.4)	0.737
Age, year	79.0 (77.0, 82.0)	82.0 (79.0, 84.0)	80.0 (77.0, 82.0)	2.0 (1.0, 3.0)	**0.001**
BMI, kg/m^2^	23.5 (21.0, 25.7)	23.4 (20.4, 25.5)	23.5 (20.8, 25.7)	– 0.2 (– 1.4, 0.9)	0.698
Education ≤ 5years	70/258 (27.1)	19/47 (40.4)	89/305 (29.2)	13.3 (– 1.7, 28.3)	0.065
Activities of daily living score	85.0 (65.0, 100.0)	45.0 (30.0, 80.0)	85.0 (60.0, 95.0)	– 30.0 (– 40.0, – 20.0)	** < 0.001**
MMSE	28.0 (26.0, 29.0)	23.0 (17.5, 27.0)	27.0 (24.0, 29.0)	– 4.0 (– 6.0, – 2.0)	** < 0.001**
Preoperative characteristics					
Hemoglobin, g/L	124.0 (112.0, 135.0)	118.0 (104.0, 132.0)	123.0 (111.0, 134.0)	– 6.0 (– 12.0, 0.0)	0.055
Anemia	66/256 (25.8)	23/50 (46.0)	89/306 (29.1)	20.2 (5.4, 35.0)	**0.004**
WBC, *10^9^/L	6.2 (5.0, 8.0)	7.1 (5.8, 8.8)	6.4 (5.0, 8.2)	0.9 (0.2, 1.6)	**0.016**
BUN, mmol/L	5.5 (4.7, 6.9)	6.4 (5.2, 8.9)	5.6 (4.8, 7.0)	1.0 (0.3, 1.7)	**0.005**
Creatinine, mmol/L	69.2 (59.7, 86.6)	67.6 (54.4, 83.8)	69.0 (58.9, 85.7)	– 3.8 (– 10.4, 2.9)	0.263
Dysaudia	48/258 (18.6)	13/49 (26.5)	61/307 (19.9)	7.9 (– 5.3, 21.2)	0.202
Visual disturbance	42/258 (16.3)	4/48 (8.3)	46/306 (15.0)	– 7.9 (– 17.0, 1.1)	0.157
Alcohol abuse	12/257 (4.7)	1/46 (2.2)	13/303 (4.3)	– 2.5 (– 7.4, 2.4)	0.404
Albumin, g/L	40.0 (36.8, 42.7)	38.2 (35.8, 39.9)	39.4 (36.5, 42.0)	– 2.0 (– 3.3, – 0.7)	**0.003**
Sleep score	16.0 (11.0, 24.0)	19.0 (12.0, 26.0)	16.0 (11.0, 25.0)	3.0 (0.0, 6.0)	0.092
Depression	24/251 (9.6)	5/40 (12.5)	29/291 (10.0)	2.9 (– 7.9, 13.8)	0.576
CCI	2.0 (1.0, 3.0)	2.5 (2.0, 3.0)	2.0 (1.0, 3.0)	1.0 (0.0, 1.0)	**0.025**
Pain NRS	142/257 (55.3)	29/46 (63.0)	171/303 (56.4)	7.8 (– 7.4, 23.0)	0.326
Operation					
Orthopedic surgery	130 (50.4)	31 (62.0)	161 (52.3)	0.622 (0.335, 1.158)	0.132
General surgery (laparoscope)	30 (11.6)	5 (10.0)	35 (11.4)	1.184 (0.436, 3.217)	0.740
General surgery (laparotomy)	41 (15.9)	8 (16.0)	49 (15.9)	1.008 (0.441, 2.304)	0.985
Urological surgery	18 (7.0)	1 (2.0)	19 (6.2)	0.272 (0.035, 2.086)	0.181
Gynecologic surgery	7 (2.7)	0	7 (2.3)	1.199 (1.140, 1.261)	0.239
Thoracic surgery	20 (7.8)	4 (8.0)	24 (7.8)	1.035 (0.338, 3.168)	0.952
Other	12 (4.7)	1 (2.0)	13 (4.2)	0.418 (0.053, 3.292)	0.393
Intraoperative characteristics					
Total intravenous anesthesia	65 (25.2)	19 (38.0)	84 (27.3)	1.820 (0.963, 3.439)	0.063
Intraoperative bleeding, ml	100.0 (50.0, 200.0)	150.0 (50.0, 300.0)	100.0 (50.0, 200.0)	20.0 (0.0, 50.0)	0.159
Blood transfusion	77 (29.8)	23 (46.0)	100 (32.5)	16.2 (1.3, 31.1)	**0.026**
Blood transfusion volume, ml	400.0 (100.0, 550.0)	400.0 (100.0, 500.0)	400.0 (100.0, 500.0)	0.0 (– 50.0, 200.0)	0.727
Dexmedetomidine	81 (31.4)	21 (42.0)	102 (33.1)	10.6 (– 4.2, 25.4)	0.145
Anesthesia during ≥ 3 h	167/256 (65.2)	25/50 (50.0)	192/306 (62.7)	– 15.2 (– 30.3, – 0.2)	**0.042**
Benzodiazepines	32 (12.4)	9 (18.0)	41 (13.3)	5.6 (– 5.8, 17.0)	0.286
Anticholinergic	39 (15.1)	8 (16.0)	47 (15.3)	0.9 (– 10.2, 11.9)	0.874
Anesthesia duration, h	3.0 (2.0, 4.5)	2.5 (2.0, 4.0)	3.0 (2.0, 4.4)	– 0.3 (– 0.5, 0.0)	0.215
Postoperative characteristics					
PCIA	73 (28.3)	8 (16.0)	81 (26.3)	– 12.3 (– 23.8, – 0.7)	0.071
Postoperative hemoglobin, g/L	115.0 (102.0, 127.0)	107.0 (92.5, 119.0)	113.5 (100.0, 126.0)	– 8.0 (– 14.0, – 2.0)	**0.006**
Postoperative anemia	114/226 (50.4)	34/48 (70.8)	148/274 (54.0)	20.4 (6.0, 34.8)	**0.010**
Postoperative WBC, *10^9^/L	10.1 (8.1, 12.4)	9.4 (7.3, 12.7)	10.1 (8.0, 12.5)	– 0.3 (– 1.5, 0.9)	0.606

*P* values were calculated by chi-square test or Mann–Whitney *U* test, where appropriate

Bold represents *P* < 0.05, with statistical difference

BIS = bispectral index, ranges from 0 to 100, with higher scores indicating more consciousness; BMI = body mass index; MMSE = mini-mental state examination, ranging from 0 to 30, with higher scores indicating better cognitive performance; WBC = white blood cell; BUN = blood urea nitrogen; CCI = Charlson comorbidity index, ranges from 0 to 33, with higher scores indicating a greater risk of long-term mortality; NRS = numeric rating scale, rate on a scale of 0 to 10, with higher scores indicating severer pain; BZD = benzodiazepines, PCIA = patient-controlled intravenous analgesia

*Differences were estimated by least square means for continuous variables

### Preoperative BIS value and delirium

Preoperative average BIS value of the delirium group was significantly lower than that of the non-delirium group [86.7 (IQR 80.0–94.0) *versus* 91.9 (IQR 89.7–95.4), *P* < 0.001] (Table 2).

**Table 2:** Preoperative BIS values

	Non-delirium (*n* = 258)	Delirium (*n* = 50)	Total (*n* = 308)	Difference*95% CI	*P*
BIS	91.9 (87.9, 95.4)	86.7 (80.0, 94.0)	91.0 (86.6, 95.2)	− 4.4 (− 7.2, − 2.0)	< 0.001

*Differences were estimated by least square means for continuous variables

**Fig. 2:**
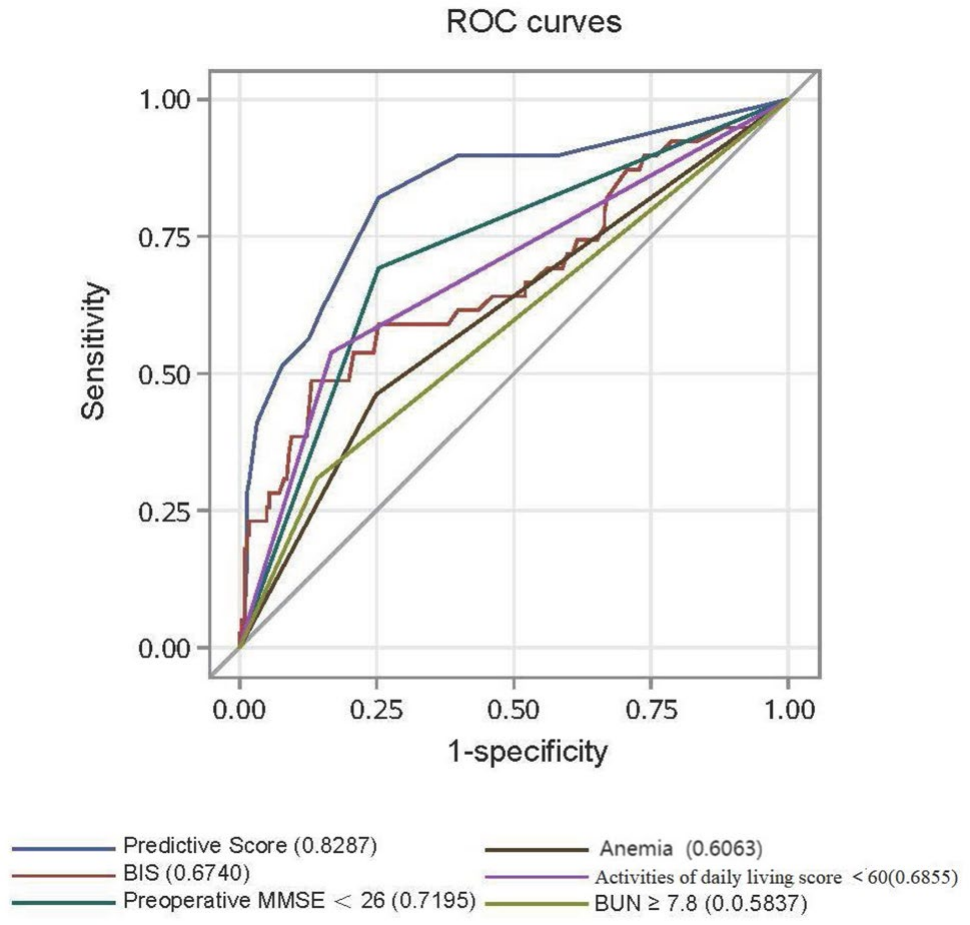
Receiver operating characteristic (ROC) curves of the predictive score

According to the ROC curve of the preoperative BIS index, the optimal cut-off value was 84, with a sensitivity of 48% and specificity of 87%. The positive predictive value and negative predictive value for forecasting POD was 43% and 89%, respectively. The area under the curve (AUC) was 0.67. The correlation between preoperative BIS and MMSE was significant *γ* = 0.234 (*P* = 0.01).Preoperative BIS, MMSE, anemia, ADL, and BUN were combined to construct the model and draw the ROC curve (Fig. 2). The AUC was 0.83, with a sensitivity of 78%, specificity of 74%, positive predictive value of 37%, and negative predictive value of 95% for forecasting POD.

### Other preoperative risk factors for delirium

According to the OR value of each risk factor in Table 3, the score corresponding to each risk factor is calculated. The top risk score was 12 and the specific definition was as following, when preoperative BIS < 85, the score was 4; when activities of daily living Score < 60, the score was 3; when preoperative MMSE < 26, the score was 3; if an anemia or high BUN (≥ 7.8 mmol/L) was coexistent, the score was 1 point. Depending on the patient's preoperative predictive score, the incidence of POD varies. As the score increases (Table4), the sensitivity decreases, and so does the negative predictive value of a new postoperative delirium, meaning that most delirium has been diagnosed.

**Table 3:** The logistic regression model of preoperative risk factors for delirium

Preoperative risk factors		Unadjusted OR (95% CI)	*P*	Adjusted OR (95% CI)	*P*	Score
BIS		0.91 (0.87–0.95)	< 0.001			
BIS < 85	No	Ref				
	Yes	7.56 (3.81–14.97)	< 0.001	5.10 (2.19–11.89)	< 0.001	4
Gender, male	Yes	1.11 (0.60–2.05)	0.737			
	No	Ref				
Age		1.13 (1.04–1.23)	0.003			
Age ≥ 80 years	No	Ref				
	Yes	2.92 (1.48–5.77)	0.002			
BMI		0.98 (0.91–1.07)	0.693			
Abnormal BMI	No	Ref				
	Yes	1.18 (0.64–2.17)	0.599			
Education ≤ 5	No	Ref				
	Yes	1.82 (0.96–3.47)	0.068			
Activities of daily living score		0.96 (0.95–0.97)	< 0.001			
Activities of daily living score < 60	No	Ref				
	Yes	6.28 (3.23–12.22)	< 0.001	3.12 (1.34–7.29)	0.008	3
Preoperative MMSE		0.83 (0.78–0.89)	< 0.001			
Preoperative MMSE < 26	No	Ref				
	Yes	5.45 (2.77–10.73)	< 0.001	3.60 (1.56–8.32)	0.003	3
Hemoglobin		0.98 (0.97–1.00)	0.060			
Anemia	No	Ref				
	Yes	2.45 (1.32–4.57)	0.005	1.62 (0.68–3.83)	0.275	1
WBC		1.18 (1.05–1.31)	0.004			
BUN		1.14 (1.03–1.26)	0.012			
WBC ≥ 10	No	Ref				
	Yes	1.85 (0.81–4.23)	0.142			
Creatinine		1.00 (0.99–1.01)	0.765			
BUN ≥ 7.8	No	Ref				
	Yes	3.13 (1.57–6.22)	0.001	1.46 (0.55–3.86)	0.444	1
Albumin		0.90 (0.84–0.97)	0.006			
Creatinine < 44 or > 106	No	Ref				
	Yes	1.75 (0.74–4.15)	0.201			
Albumin < 35	No	Ref				
	Yes	1.84 (0.86–3.97)	0.118			
Dysaudia	No	Ref				
	Yes	1.58 (0.78–3.21)	0.205			
Visual disturbance	No	Ref				
	Yes	0.47 (0.16–1.37)	0.166			
Alcohol abuse	No	Ref				
	Yes	0.45 (0.06–3.58)	0.453			
Preoperative sleep score		1.03 (0.99–1.07)	0.127			
Discharge activity score		0.96 (0.95–0.98)	< 0.001			
Preoperative depression	No	Ref				
	Yes	1.35 (0.48–3.77)	0.565			
CCI		1.23 (1.02–1.48)	0.027			
Preoperative pain NRS	No	Ref				
	Yes	1.38 (0.72–2.64)	0.328			

OR and 95% CI were estimated by the logistic model. The score was calculated from the regression coefficient. ln (OR) = regression coefficient. We set the risk coefficient 0.378 (BUN ≥ 7.8) as the benchmark coefficient, which was recorded as 1 point. For example, ln (5.10) = 1.629 (BIS < 85), 1.629/0.378≈4, when preoperative BIS < 85, the score was 4

**Table 4:** Accuracy, positive and negative predictive values for delirium across different score thresholds

Score	Sensitivity (95% CI)	Specificity (95% CI)	PPV (95% CI)	NPV (95% CI)
0	1.00 (1.00–1.00)	0.00 (0.00–0.00)	0.16 (0.12–0.20)	–
1	0.90 (0.82–0.98)	0.43 (0.37–0.49)	0.23 (0.17–0.29)	0.96 (0.92–0.99)
2	0.90 (0.82–0.98)	0.59 (0.53–0.65)	0.30 (0.22–0.37)	0.97 (0.94–1.00)
3	0.90 (0.82–0.98)	0.60 (0.54–0.66)	0.31 (0.23–0.38)	0.97 (0.94–1.00)
4	0.78 (0.67–0.89)	0.74 (0.69–0.79)	0.37 (0.28–0.46)	0.95 (0.91–0.98)
5	0.60 (0.46–0.74)	0.86 (0.82–0.90)	0.45 (0.33–0.57)	0.92 (0.88–0.95)
6	0.50 (0.36–0.64)	0.89 (0.85–0.93)	0.46 (0.33–0.60)	0.90 (0.86–0.94)
7	0.44 (0.30–0.58)	0.93 (0.90–0.96)	0.56 (0.41–0.72)	0.90 (0.86–0.93)
8	0.36 (0.23–0.49)	0.97 (0.95–0.99)	0.72 (0.54–0.90)	0.89 (0.85–0.92)
9	0.26 (0.14–0.38)	0.98 (0.96–1.00)	0.76 (0.56–0.97)	0.87 (0.83–0.91)
10	0.22 (0.11–0.33)	0.99 (0.98–1.00)	0.79 (0.57–1.00)	0.87 (0.83–0.91)
11	0.12 (0.03–0.21)	0.99 (0.98–1.00)	0.67 (0.36–0.97)	0.85 (0.81–0.89)
12	0.04 (0.00–0.09)	0.99 (0.98–1.00)	0.50 (0.01–0.99)	0.84 (0.80–0.88)

Sensitivity, specificity, PPV, and NPV were estimated by the logistic model

CI, confidence interval. PPV, positive predictive value. NPV, negative predictive value

**Fig. 3:**
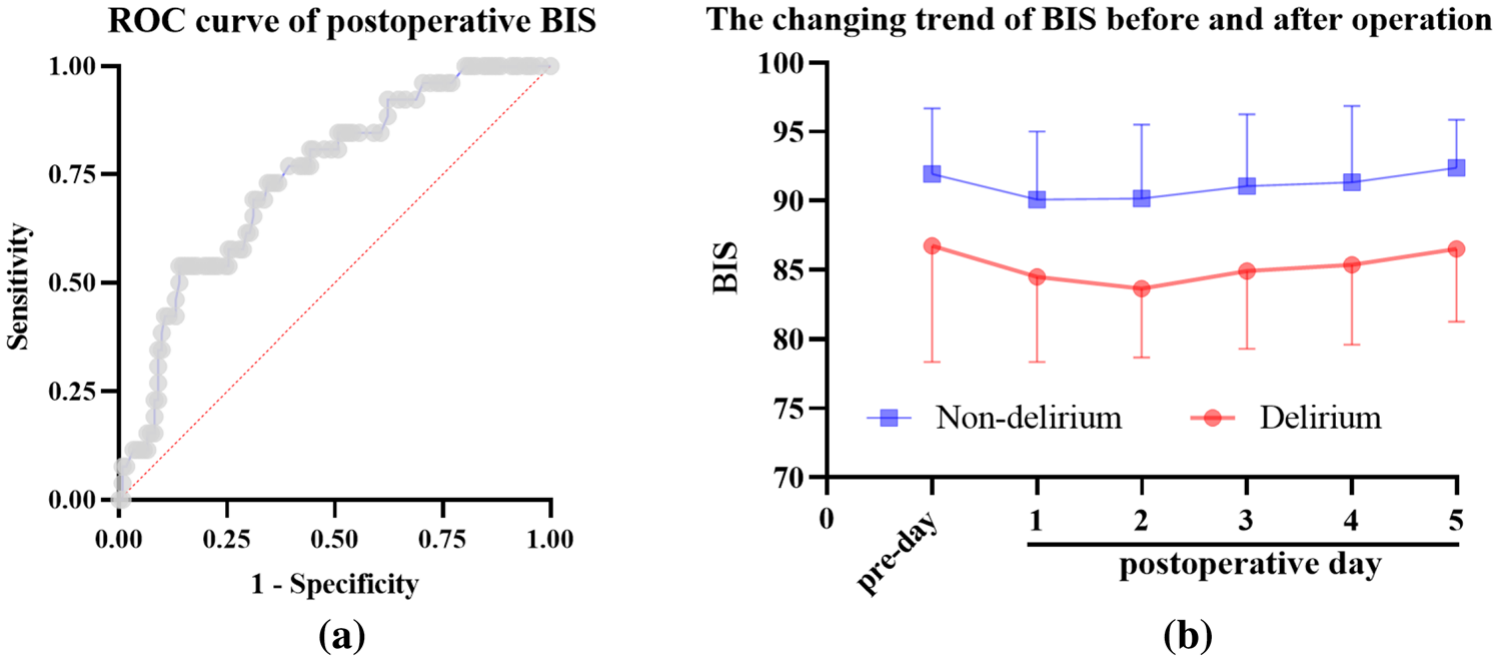
**a** ROC curves of the first postoperative day BIS. **b** BIS trend chart of the day before and 5 days after the operation

According to the ROC curve of the postoperative (the right day after surgery) BIS index, the optimal cut-off value was 84, with a sensitivity of 54% and specificity 75% for diagnosis POD and area under curve was 0.74 (Fig. 3A). To analyze the changes of BIS after surgery, the daily average of BIS after surgery is shown in Fig. 3B. The average BIS of patients with delirium 5 days after surgery was lower than that of patients without delirium, and the lowest BIS was found on the second day after surgery (83.6 versus 90.2).

## Discussion

Recent evidence has described the risk factors associated with postoperative delirium [1, 15]. Nevertheless, the delirium prediction tool has not been quantified, and we hope to obtain an objective preoperative delirium prediction tool through this study to provide the reference for clinical practice. In this study, delirium occurred in 50 of the 308 (16.2%) patients, which was in line with the incidence of 11–46% reported in previous studies [1].

During general anesthesia, BIS monitoring facilitates anesthetic titration and reduces episodes of deep levels of anesthesia [16]. Moreover, depth of anesthesia should be monitored in all patients aged over 60 years [17]. Several studies have clearly demonstrated the advantages of optimization of anesthesia depth (bispectral index between 40 and 60) as a pragmatic interventions to reduce postoperative cognitive impairment [18, 19].

The cerebral electrical bioimpedance of patients with brain injury is different from that of healthy people [20], and related studies have shown that the cerebral electrical bioimpedance of patients with chronic stroke is different [21, 22], which may be the reason for the measurement of BIS data in patients. The BIS was lower in patients with dementia compared with those without [23]. If a pathophysiological condition that converts brain state, such as the metabolic balance, the electroencephalogram could be changed, and then the value of BIS will be changed correspondingly. This is the basis for the idea that BIS is associated with this neurological functional change in the development of delirium.

In this study, preoperative BIS, as a single predictor, showed high specificity and low sensitivity to the prediction of POD, with an area under the ROC reaching 0.67. Its high specificity could be used as a clinical index to exclude those low delirium risk patients. Its low sensitivity may be related to the pathology of delirium (usually caused by a variety of factors). When preoperative BIS, MMSE, anemia, ADL, and BUN were combined to establish the predictive model, the sensitivity and specificity were much higher, indicating that a multi-factor model might be a better predictive tool for POD.

The mean BIS of delirious patients after surgery within 5 days was lower than that of patients without delirium. On the second postoperative day, the difference value of BIS between the delirious and non-delirious patients reached its maximum (83.6 versus 90.2), which was consistent with a phenomenon that delirious symptoms always occurred in the first two postoperative days.

MMSE is a standardized tool for assessing mental states and was first used in 1975, which involves orientation, attention, immediate and short-term recall, language, and the ability to follow simple verbal and written commands [24]. MMSE scale is the most commonly used cognitive function screening scale in clinical practice [25]. The cut-off value of dementia screening in the population with a primary education level or above was ≤ 26 [11, 26]. In the current study, MMSE was used to assess the cognitive function, and it was found that lower MMSE score always accompanied by a higher incidence of delirium, suggesting the preoperative cognitive dysfunction would contribute to the development of POD [27, 28]. In this study, we found higher preoperative BUN was accompanied by a higher incidence of delirium, which is consistent with previous study [29]. Its pathogenesis may be related to neurotoxin [30]. Moreover, we found the preoperative anemia and low ADL were risk factors of POD, which supported the view that preoperative cognitive function and activities of daily life were strongly associated with the outcome of patients after surgeries [31], 32. In summary, the prevention of POD should contain multi-factors, such as cognition training, anemia correcting, promoting activities of daily life, and improving body homeostasis.

The study also had the several limitations. First, a feature of raw EEG might be a better predictor than the BIS value, such as the time–frequency and power spectrum of EEG. However, our aim of this study was find a more convenient index to screen out patients with high risk of delirium before surgery. Second, this is a single-center study and so the conclusion needs further confirmation.

## Conclusion

BIS in delirium patients aged over 75 was lower than that in non-delirium patients before and after non-neurosurgery and non-cardiac surgery. The model of integrating BIS, mini-mental state examination, anemia, activities of daily living, and blood urea nitrogen is a promising tool for predicting postoperative delirium in patients aged over 75.

## Supplementary Information

Below is the link to the electronic supplementary material.

Supplementary file1 (TEX 41 KB)

## Data Availability

The authors confirm that the data supporting the findings of this study are available within the article.
